# The Wavelength-Dependent SERS Template Based on a Nanopillar Array

**DOI:** 10.3390/ma15217446

**Published:** 2022-10-24

**Authors:** Jiayi Li, Rui Li, Ying Xu, Xiaojun Xue, Xiaoming Chen, Hsiang-Chen Chui

**Affiliations:** 1 School of Optoelectronic Engineering and Instrumentation Science, Dalian University of Technology, Dalian 116024, China; 2 College of Physics, Dalian University of Technology, Dalian 116024, China; 3 PipeChina Group, Beijing Pipe Co., Ltd., Beijing 100020, China; 4 Department of Photonics, National Cheng Kung University, Tainan 70101, Taiwan

**Keywords:** nanostructure, surface-enhanced Raman scattering, wavelength-dependent

## Abstract

Surface-enhanced Raman spectroscopy (SERS) can be regarded as a powerful tool for probing chemical molecules by effectively enhancing Raman signals. However, the enhancement factors depend on the SERS template, the probed molecular structures, and the excitation laser wavelength. Herein, we proposed a simple and easily fabricated nanostructured template for SERS and analyzed the wavelength-dependent factors. Three types of golden nanopillar arrays on silicon wafers were designed and manufactured. The SERS signals of the Rhodamine 6G (R6G) molecules were extracted. Three laser sources, a blue 17 mW 458 nm diode laser, a green 20 mW 532 nm laser, and a red 6 mW 633 nm laser, were employed as the excitation laser sources. The 458 nm laser was located far from the resonate spectrum of R6G. The optical intensity distributions for the different SERS templates excited by three laser beams were also simulated. The enhancement factors (EFs) of R6G on the three nanostructured templates were measured and compared. The photoluminescence spectrum of the nanostructured templates and SERS signals of R6G were also measured. In addition, the experimental results concerned optical simulations. The analysis tool that was used was a convolution profile of multiple Lorentzian line shapes with a Gaussian profile. It is helpful to understand the SERS signals when the excitation laser wavelength is located out of the resonance region of molecules. It can also provide a new design approach to fabricate an SERS Template with a nanopillar array for different excitation wavelengths.

## 1. Introduction

Surface-enhanced Raman-scattering (SERS) spectroscopy is a powerful analytical tool and has vast potential applications in many fields such as chemical and biological sensing [1] due to the process of Raman Signal enhancement. In 1974, M. Fleischmann observed the significant Raman scattering of pyridine on coarse-silver for the first time [2]. Subsequently, George C. Schatz [3] and Lasse Jensen [4] described the electromagnetic enhancement mechanism and chemical enhancement mechanism, respectively.

The SERS substrate is the most important factor, and it can determine the intensity of SERS signals. In the past, the choice of the SERS substrate included nanoparticle (NPs) materials [5,6] or three-dimensional (3D) structures [7,8]. Herrera et al. [9] achieved an excellent SERS result by using gold and silver NPs prepared by laser ablation as the substrate. Freeman et al. [10] used monodisperse gold and silver colloidal NPs to self-assemble monolayers on polymer-coating substrates to generate macroscopic surfaces, which are highly active for SERS. Sanchez-Iglesias et al. [11] used block copolymer micelle nanolithography to generate arrays of Ag NPs containing high-density hot spots. The degree of SERS enhanced by NPs depends on many factors, such as the size, shape, and material of the NPs [12,13]. Three-dimensionally structured SERS substrates have been fabricated with Ag or Au films. Different types of 3D structures can be designed, such as Ag-decorated Si nanocone arrays [14], Ag nanosheet-assembled micro-hemispheres [15], and Ag NP-grafted PAN-nanohump array films [16]. The fabrication processes include electron-beam lithography [17], integrating photolithographic microstructures and self-assembly [18], soft lithography, and nano-sphere lithography [19]. Other 3D structures have been reported, including regular hexagonal arrays [20], 3D flower-like gold microstructures [21], and Au-coated ZnO nanorods [22].

In the consequent studies, some materials had some unique advantages when they were used as SERS substrates [23,24,25,26]. Two-dimensional (2D) materials have increasingly become research objects due to their excellent performance [27,28,29]. In addition, non-novel metallic NPs or nanostructures have also been used in SERS applications, such as semiconductor nanostructures [30] and carbon nanostructures [31]. To date, the SERS substrates have had broad application prospects in biomedicine [32,33], art protection [34], molecular detection [35,36], food safety [37], and other fields.

In this work, we proposed a simple and easily fabricated nanostructured template for SERS. The three types of golden nanopillar arrays on silicon wafers were designed and fabricated. We extracted the photoluminescence (PL) spectra and the SERS signals of Rhodamine 6G (R6G) molecules on these nanostructured templates produced by a 458 nm laser, a 532 nm laser, and a 633 nm laser. In addition, the optical intensity distributions for the different SERS templates excited by the three laser beams were simulated using the finite-difference-time-domain (FDTD) method according to Maxwell’s equations. Eventually, we investigated the sample effect for the enhancement factors (EFs). R6G is a cationic dye with strong absorption in the visible and a high fluorescence yield. When R6G molecules with an SERS substrate are excited with visible light, they show a molecular resonance Raman effect in addition to the SERS effect that comes from the localized surface plasmon resonance (LSPR) [38]. The SERS performance is largely dependent on the LSPR of the substrate at a given excitation wavelength. The laser excitation at 458 nm has the best SERS performance for the substrate specially designed [39].

## 2. Materials and Methods

### 2.1. Sample Preparations

**Figure 1:**
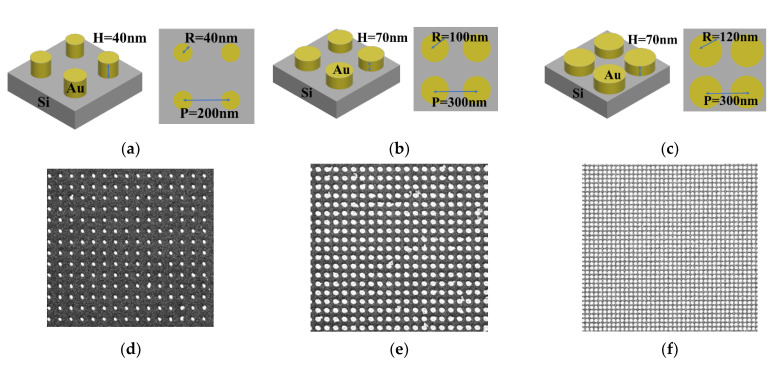
(**a**) The design of R40 sample, (**b**) the structure of R100 sample, (**c**) the structure of R120 sample, (**d**) the SEM images of R40 sample, (**e**) the SEM images of R100 sample, and (**f**) the SEM images of R120 sample.

The nanostructured templates were designed as a nanopillar array with three different nanopillar sizes on silicon wafer. The radius and the height of the nanopillars and the pitch between two nanopillars of the nanostructured templates are shown in Figure 1a–c. The samples were named according to the nanopillar sizes, e.g., the R40 sample, as shown in Figure 1a, corresponds to the 40 nm radius nanopillars. The R100 and R120 samples were also named by this rule. The samples were prepared by an electron beam evaporation system and an electron beam writer (ELS-7500EX, Elionix Inc., Tokyo, Japan). These samples were prepared by Center for Micro/Nano Science and Technology, National Cheng Kung University. The SEM images for the SERS templates are shown in Figure 1d–f.

At the same time, 500 mL 10^−3^ M R6G solutions were prepared. The 0.24 g R6G powder was dissolved in a 10 mL of 99% alcohol, and then diluted in 500 mL of deionized water. In addition, three types of samples were soaked in the solution for 1 h. The Raman spectra were measured immediately after the samples were removed from the solution.

### 2.2. Micro Raman Spectroscopy System

The optical layout of Raman system is a confocal microscopy configuration with a 17 mW 458 nm diode laser, a 19 mW 532 nm frequency-doubling Nd: YAG laser, and a 6 mW 633 nm He-Ne Laser. A laser beam was focused by a 100 X objective lens (NA = 0.9) on the sample plate with a 2 μm spot size. A high-resolution spectrometer (Jobin Yon iHR550, HORIBA Ltd., Tokyo, Japan) was utilized to collect the Raman signals. The spectral resolution was 0.7 cm^−1^. The integration time constant was 0.5 s. The lateral spatial resolution was estimated was 0.7 μm, and the axial spatial resolution was calculated as 4.2 μm.

### 2.3. The Simluations of the Optical Intensity Distributions

The related research works regarding the SERS enhancement mechanism were reported [40,41,42,43]. Typically, the researchers have focused on its surface roughness. However, as a uniform property of the reflective surface, the electromagnetic (EM) field enhancement effect generated by the surface roughness is also even in the SERS plane. The local strong enhancement effect in a certain area cannot be defined. These designed nanopillar structures can provide large, strong EM field enhancement around the nanostructured area and boost the generations of the SERS signals. So, the optical intensity distributions for the different SERS templates excited by three laser beams were simulated using FDTD method according to Maxwell’s equations.

## 3. Experimental Results

### 3.1. Photoluminescence Spectra

**Figure 2:**
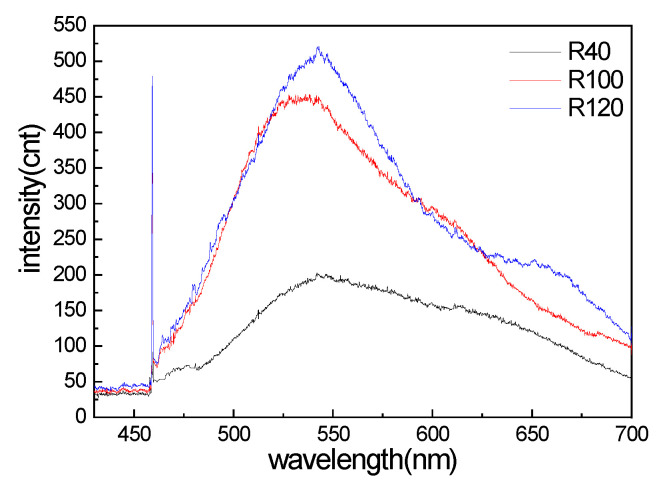
The photoluminescence spectrum of R6G on three nanostructured templates (laser beam is 458 nm).

The PL spectra of R6G on the three nanostructured templates are shown in Figure 2. The PL peak was found around 547 nm. Compared with the PL signal intensities, the Raman signals could be estimated on the order of −3 to −6 times lower than the PL signals. The 458 nm laser excitation can excite the unique Raman spectrum, which is located far from the PL spectrum around 547 nm.

### 3.2. The SERS Signals

**Figure 3:**
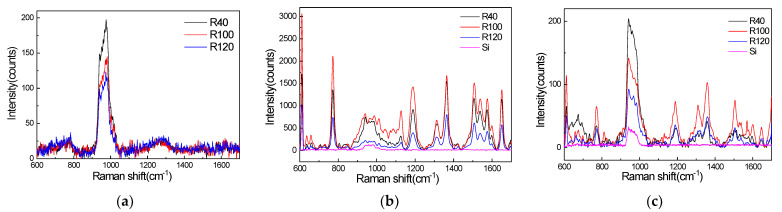
(**a**) SERS signals of R6G adsorbed on three structures, which were excited by the 458 nm laser; (**b**) SERS signals of R6G adsorbed on four structures, which were excited by the 532 nm laser; (**c**) SERS signals of R6G adsorbed on four structures, which were excited by the 633 nm laser.

Figure 3 shows that the SERS signals of R6G on the three nanostructured templates and a silicon wafer were excited by a 458 nm laser, 532 nm laser, and a 633 nm laser. Shown in Figure 3a, we compared the peak height and peak position of SERS signals produced by the 458 nm laser and concluded that the R40 sample exhibits the greatest enhancement of the SERS signal of R6G. In addition, weaker enhancement factors were obtained for the R100 and R120 samples. As shown in Figure 3b, the SERS signals produced by the 532 nm laser have a strong intensity, and the R100 sample has the greatest enhancement, while the R120 sample has the worst enhancement. The SERS signals excited by the 633 nm laser are shown in Figure 3c. The R40 nanopillar array has the greatest enhancement at the peak at 772 cm^−1^, and the R100 nanopillar array has the greatest enhancement at the other peaks.

## 4. Results and Discussions

### 4.1. The Light Intensity Distribution Due to the SERS Templates

**Figure 4:**
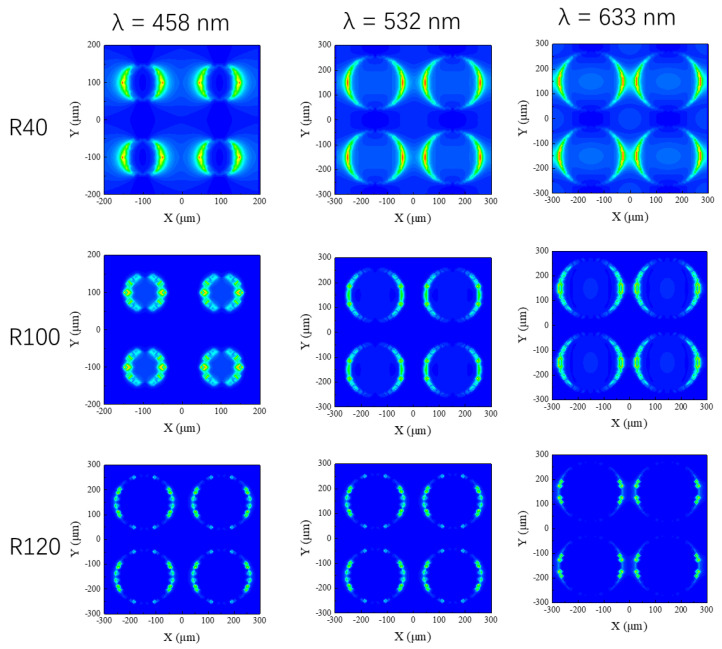
The optical intensity distributions for different SERS templates (R40, R100, and R120) under three excitation wavelengths (λ = 458 nm, 532 nm, and 633 nm).

In order to verify this enhancement effect with respect to the SERS templates, simulation procedures were performed using the FDTD method according to Maxwell’s equations. The optical intensity distributions for the different SERS templates (R40, R100, and R120) under three excitation wavelengths (λ = 458 nm, 532 nm, and 633 nm) are shown in Figure 4. One can observe that the three nanostructured SERS templates also have better enhancement at a short wavelength (λ = 458 nm). Comparing the enhancement effect of the three nanostructured SERS templates at the same wavelength, one can find that the EM field enhancement effect of R40 at the three wavelengths is the strongest, and the EM field enhancement effects of R100 and R120 are almost the same, which matches the experimental data in which the R40 SERS template has the best enhancement effect on the R6G signals.

### 4.2. Linewidth Analysis

From the Raman spectra excited by the 458 nm laser, we found a significant peak in the range of the Raman shift from 840 cm^−1^ to 1060 cm^−1^, in which the peak is apparently superimposed by multiple peaks. This significant peak could also be found when the samples were excited by the 532 nm and 633 nm lasers, which is in accordance with the results reported by Tan et al. [39]. We tried to fit and decompose this overlapping peak signal and obtain a few peaks of different Raman shifts. Further analysis was achieved by comparing the intensity of each peak of different types of Au nanopillar arrays.

**Figure 5:**
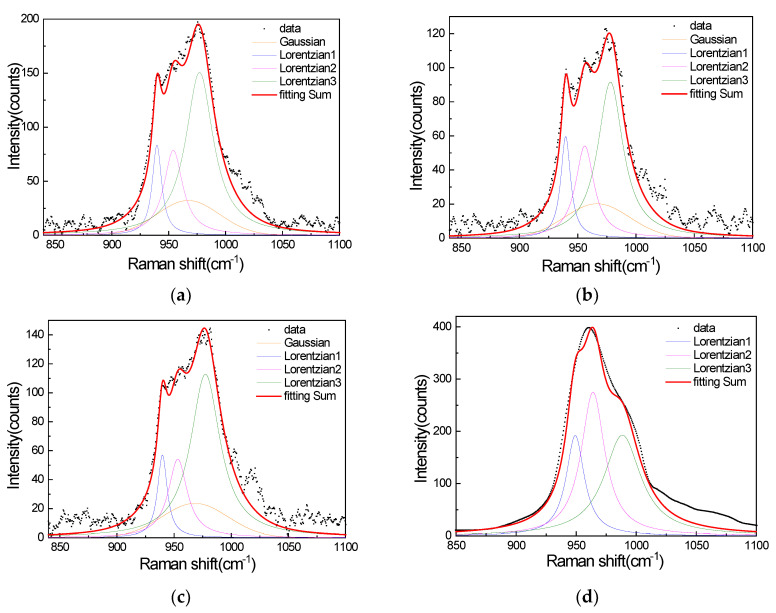
(**a**) SERS signals of R40 with three Lorentzian line shapes and a Gaussian, (**b**) SERS signals of R100 with three Lorentzian line shapes and a Gaussian, (**c**) SERS signals of R120 with three Lorentzian line shapes and a Gaussian, and (**d**) the Raman spectrum of R6G on a silicon wafer with three Lorentzian line shapes.

The Peakfit program embedded inside MATLAB was chosen here. The Peakfit algorithm relies on the first derivative to find peaks and resolve signals [5]. Using the Peakfit algorithm, the SERS spectra of the performed background subtraction were fitted to three Lorentzian line shapes and a Gaussian, and the Raman spectrum of R6G on a silicon wafer was fitted to three Lorentzian line shapes. The result of the fitting is shown in Figure 5a–d.

**Figure 6:**
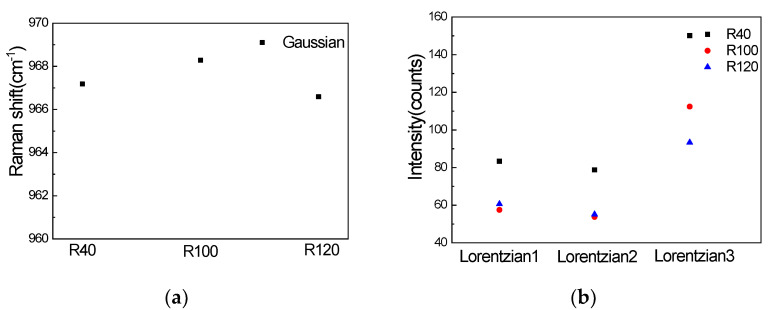
(**a**) Gaussian peak positions of three types of nanopillar array; (**b**) peak height of three fitting Lorentzian profiles.

Comparing the peak positions of the Gaussian signal, which are shown in Figure 6a, it can be concluded that under the same laser (458 nm) and other conditions, the difference in the nanopillar array will not affect the peak positions of the Gaussian signal. Figure 6b shows the peak height of the three fitted Lorentzian profiles. By comparing the peak heights of the three nanopillar arrays, we can see that the intensity of the three peak heights of R40 is the strongest, the intensity of Lorentzian1 and Lorentzian2 of R100 and R120 are almost the same, and the intensity of Lorentzian3 of R100 is stronger than R120.

### 4.3. Enhancement Factors

To quantify the *SERS* enhancement using the three samples, the peak at 772 cm^−1^ was used to calculate the *SERS EF*s using the following equation:

(1) $$EF=\frac{I_{SERS}}{I_{NR}}$$

where *I_NR_* and *I_SERS_* are the peak intensities for the Raman spectra of R6G from the silicon wafer and the nanostructured template.

**Figure 7:**
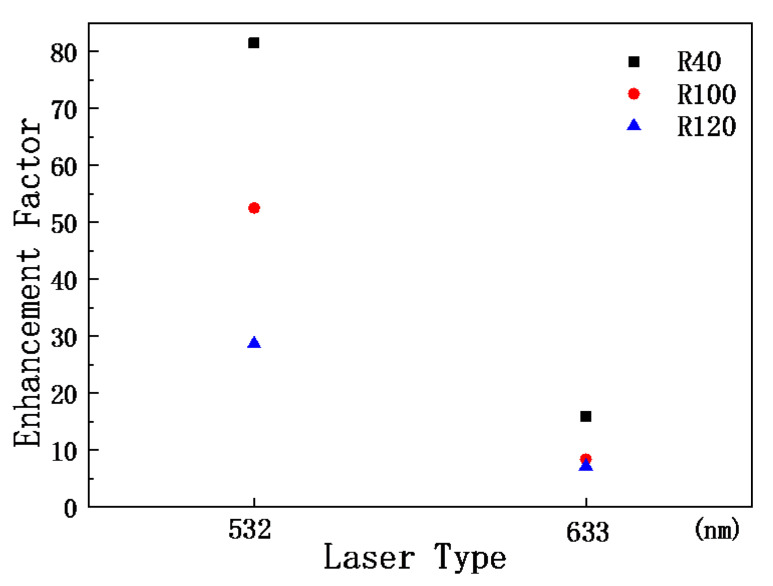
The enhancement factors 772 cm^−1^ peak of R6G on three nanostructured templates.

Figure 7 shows the EFs of three nanostructures at 772 cm^−1^. By comparing the *EF*s, it can be determined that the R100 nanopillar array has the greatest enhancement with respect to the Raman signal of R6G.

## 5. Conclusions

In this work, the SERS signals of R6G adsorbed on three nanostructured templates and a silicon wafer were measured, which were excited by the 458 nm, 532 nm, and 633 nm laser beams. The photoluminescence spectra of R6G on the three samples produced by a 458 nm laser beam were recorded. The EFs of the three nanostructures at 772 cm^−1^ were obtained. Comparing the intensity of the SERS signals, the R40 sample has the strongest signal excited by the 458 nm laser, the R100 sample has the strongest signal excited by the 532 nm laser, and the R40 sample has the strongest signal excited by the 633 nm laser. The EF of the R40 sample is the maximum among the three nanostructured templates. The SERS spectra of the performed background subtraction were fitted to three Lorentzian line shapes and a Gaussian. The difference in the nanopillar array has no effect on the peak positions of the Gaussian signal. The optical measurements of the SERS signal enhancements using our designed SERS templates for other dyes will be performed soon. The enhancement factors and the limit-of-detection values for various concentrations with the R6G dye will also be investigated. Nanostructured templates at 785 nm for biological samples will be prepared in the near future. The proposed nanostructured template can be applied to biological samples and this research is ongoing.

## Data Availability

The data presented in this study are available on request from the corresponding author.
